# Experimental Study of Key Effect Factors and Simulation on Oil Displacement Efficiency for a Novel Modified Polymer BD-HMHEC

**DOI:** 10.1038/s41598-018-22259-z

**Published:** 2018-03-01

**Authors:** Chao Wang, Pengcheng Liu, Yanling Wang, Zhe Yuan, Zhenhua Xu

**Affiliations:** 1 School of Energy Resources, China University of Geosciences, 29 Xueyuan Road, Beijing, 100083 China; 2 School of Petroleum Engineering, Petroleum University of China, 66 Changjiang West Road, Qingdao, 266555 China

## Abstract

A novel synthetic hydrophobically modified hydroxyethyl cellulose (HEC) using bromododecane (BD) was developed in our previous paper, which we denote as BD-HMHEC. A series of one dimensional core displacement experiments were continually conducted to evaluate the key effect factors on the resistance factor (*F*_R_) and residual resistance factor (*F*_RR_) of BD-HMHEC solution, including polymer concentration, core permeability and injection rate. Results have shown that BD-HMHEC has higher *F*_R_ and *F*_RR_ and has much better oil displacement performance than HEC during oil displacement process. Meanwhile, compared with HEC flooding, the key effects on oil displacement efficiency of BD-HMHEC flooding were investigated, including polymer concentration, injection slug and injection rate. A numerical simulation study has been developed by the Computer Modelling Group (CMG) simulator. Results have shown that BD-HMHEC flooding could cause better oil displacement efficiency than HEC flooding at the same condition. As indicated by one dimensional core displacement experimental results, the further incremental oil recovery of switching to BD-HMHEC flooding could increase by 7.0~8.0% after hydrolyzed polyacryamide (HPAM) flooding. The studies indicate that BD-HMHEC has great potential application during enhanced oil recovery (EOR) processes in oilfields.

## Introduction

Enhanced oil recovery (EOR) techniques have been verified as effective oil development techniques in oilfields where conventional methods failed or were unfeasible^1,2^. Polymer flooding, which belongs to a kind of chemical EOR method, is one of good candidates during chemical processes that are designed to be used massively in EOR following water flooding^3,4^. The limited number of available commercial polymers currently employed has been the subject of recent developments aimed at improving their performances in EOR. Indeed, an alternative concept has been studied in recent decades^5,6^.

Unlike the conventional water-soluble polymers, i.e. partially hydrolyzed polyacrylamide (HPAM) in oilfields, when hydrophobically modified water-soluble polymer (HM-polymer) is dissolved in water, supramolecular aggregates and the reversible network structures are formed owing to association among the hydrophobic groups, thus, the viscosity of the solution increases significantly^7–9^. HM-polymers usually have special rheological properties such as better thickening, thermal-resistance, salt-tolerance, shear-resistance, and acid/alkali-resistance^10–13^.

Many different monomers and hydrophobic monomers have been used to prepare acrylamide based on HM-polymers by free radical polymerization^10–17^. Hydrophobically modified hydroxyethyl cellulose (HM-HEC) is the most significant type, which is prepared by hydroxyethyl cellulose (HEC) by reaction with alkyl halides, acid halides, acid anhydrides, isocyanates, or epoxides^1,5,18,19^. The synthesis, characterization, stability and rheological properties of associated cellulosic thickeners have been intensively studied, which shows that HM-HEC has much better thickening ability and stronger strain hardening behavior than its parent (hydroxyethyl cellulose)^12,20–29^.

In previous work, the synthetic HM-HEC by the macromolecular reaction between HEC and the long chain alkyl halides of bromododecane (BD) was developed, herein denoted as BD-HMHEC^30,31^. The optimum condition of synthesis and oil displacement mechanism was only discussed. But, its oil displacement performance and corresponding efficiency were not clearly evaluated. Meanwhile, the production parameters as a good oil displacement system to enhance oil recovery were not touched. In this study, a series of laboratory experiments were conducted to investigate the key effects on the resistance factor (*F*_R_) and residual resistance factor (*F*_RR_) of BD-HMHEC solution in contrast with HEC solution, including polymer concentration, core permeability and injection rate. Furthermore, compared with HEC flooding, the key effects on oil displacement efficiency of BD-HMHEC flooding were investigated, including polymer concentration, injection slug and injection rate. Thirdly, in order to verify the different effects on HEC flooding and BD-HMHEC flooding, the paper evaluated the oil displacement performance by Computer Modelling Group (CMG) simulator. One dimensional core displacement experiments were conducted to further investigate the oil displacement efficiency of BD-HMHEC solution following conventional polymer flooding (e.g. HPAM) and water flooding at different permeability cores.

## Experiment and Simulation

### Experiments of *F*_R_ and *F*_RR_ measurement

#### Methodology

The resistance factor (*F*_*R*_) and the residual resistance factor (*F*_*RR*_) are the most important parameters for polymer flooding, which can determine the oil displacement efficiency in oil field development.

The polymer resistance factor (*F*_*R*_) of a given fluid is the mobility ratio of the brine and polymer. Residual resistance factor (*F*_RR_) of a given fluid refers to the ratio of the brine permeability before and after polymer solution flows through the core.

The resistance factor (*F*_*R*_) refers to the mobility ratio of fluid, which can be expressed by Eq. (1)

1 $${F}_{R}=\frac{{\lambda }_{w}}{{\lambda }_{p}}=\frac{{k}_{w}}{{\mu }_{w}}/\frac{{k}_{w}}{{\mu }_{p}}$$

Based on Darcy’s Law, *F*_R_ could be written as follows:

2 $${F}_{R}=\frac{{k}_{w}}{{\mu }_{w}}/\frac{QL}{A{\rm{\Delta }}p}\times {10}^{-1}$$

Residual resistance factor (*F*_*RR*_) refers to the ratio of the brine permeability before and after polymer solution flows through the core, which is expressed as,

3 $${F}_{RR}=\frac{{k}_{wi}}{{k}_{wa}}$$

Where, *F*_*R*_ *is* resistance factor, *f*; *F*_*RR*_ is residual resistance factor, *f*; *λ*_w_ is brine mobility, μm^2^/(mPa·s); *λ*_*p*_ is polymer solution mobility, μm^2^/(mPa·s); *k*_w_ is effective permeability of brine,um^2^; *k*_p_ is effective permeability of polymer solution, um^2^; *u*_w_ is viscosity of brine, mPa.s; *u*_p_ is viscosity of polymer solution, mPa.s; *Q* is flow rate through the core, cm^3^/s; *L* is length of the core, cm; *A* is the section area of the core, cm^2^; Δ*p* is pressure difference between two ends of the core, atm; *k*_wi_ is brine permeability before polymer flows through the core, um^2^; *k*_wa_ is brine permeability after polymer flows through the core, um^2^; *k* is pore permeability,um^2^.

*F*_*R*_ reflects the capacity of mobility reduction by polymer flooding, and *F*_*RR*_ reflects the permeability reduction caused by polymer flooding. Their values are always greater than 1.0 which results in better oil sweep efficiency. The larger *F*_*R*_ and *F*_RR_ mean more potential to improve the sweep efficiency and higher incremental oil recovery by polymer flooding.

#### Experimental procedure

**Figure 1:**
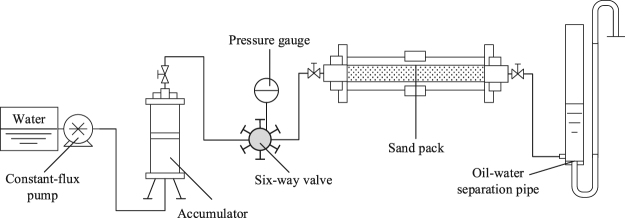
Schematic of experimental apparatus for *F*_R_ and *F*_RR_ measurement and oil displacement.

Figure 1 shows a schematic drawing of the core displacement set-up to measure resistance factor (*F*_R_) and residual resistance factor (*F*_RR_). The experimental procedures were described as below^31^.

1. The sand-pack tube was packed with the formation sand. The core was saturated with the formation water, aged for about 4.0 h.
2. The formation water was injected into the core at a certain rate. The pressure drop across the cores was measured simultaneously, and substituted into Darcy’s law to calculate the permeability. Then, the sand-pack tube was weighed and its porosity was measured.
3. Polymer solution was injected until the pressures between two ends of the core were stable. *F*_*R*_ was measured based on Eq. (2).
4. After that followed the formation water which was injected until the pressure drop across the core was stable again; *F*_*RR*_ was measured based on Eq. (3).

### Experiments of oil displacement efficiency

1. The sand-pack tube was filled with formation sand, and its weight was measured.
2. The tube was saturated using the formation water (aged for about 4.0 hours), and the water was injected. The weight of the sand-pack tube, permeability and porosity were measured or calculated.
3. Crude oil was injected into the tube until the water was no longer produced. Irreducible water saturation was calculated and the tube was aged at 45.0 °C for 24.0 h.
4. Water was injected at a certain rate until the water cut reached 98.0%, whereas, the oil recovery by water flooding was calculated.
5. Different injection slugs of BD-HMHEC or HEC solutions were injected at a certain rate until the water cut exceeded 98%, while the incremental oil recovery by polymer flooding was calculated.
6. Different injection slugs of HAPM solutions were injected at a certain rate, afterwards, water flooding was switched until the water cut was 98.0%, hence, and the oil recovery by HAPM flooding was calculated.
7. Then, different injection slugs of BD-HMHEC solutions were injected at a certain rate until the water cut exceeded 98%, while the incremental oil recovery by BD-HMHEC flooding was calculated.

### Simulation verification of oil displacement efficiency

**Figure 2:**
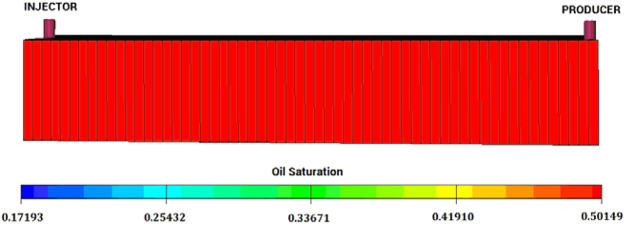
The grid blocks arranged of one dimensional core model for HEC and BD-HMHEC simulation verification.

In order to verify the different effects on HEC and BD-HMHEC flooding processes. Computer Modelling Group (CMG) (Canada) software was employed to evaluate their corresponding oil displacement efficiencies. The software is a compositional hydrocarbon reservoir simulator which is a very useful tool to model multi-chemical compositions flooding. Figure 2 shows the primary assumptions were listed as below based on the one dimensional core model in the laboratory^32–34^.

1. A base model was developed and used by water flooding. Then this model was changed and used in modeling HEC and BD-HMHEC flooding after the above water flooding.
2. A 2-spot well spacing was used with one producer and one injector placed on opposite of the two sides in core. The model had one layer, which was horizontal, homogeneous and isotropic with uniform thickness; the fluids presented were only oil and water and the effects of capillary pressure were neglected.
3. The grid blocks describing the X-, Y-, Z- directions were 60 × 1 × 1 and described a 4.676 cm^2^ area of 30 cm in the X-direction, 1.22 cm in the Y-direction and 1.22 cm in the Z-direction. The injector was placed respectively in the cell (1, 1, 1) and the producer in the cell (60, 1, 1) with both penetrated in the grid blocks in vertical direction.
4. The injector was injected at a constant rate (0.5 mL/min) and the producer was allowed to flow at constant bottomhole flowing pressure (0.1 MPa) according to the experimental core flood tests conditions.

## Results and Discussion

### Effect factors on *F*_R_ and *F*_RR_ of BD-HMHEC

In order to evaluate the thickening viscosity of BD-HMHEC solution, the experiments were conducted to evaluate the key effect factors (including polymer concentration, core permeability and injection rate) on the resistance factor (*F*_R_) and residual resistance factor (*F*_RR_) of HEC and BD-HMHEC solutions.

#### Effect of polymer concentration

In order to evaluate the effect factors on *F*_R_ and *F*_RR_, the polymer concentration of 2000 mg/L, 3000 mg/L, 4000 mg/L, 5000 mg/L and 6000 mg/L were selected respectively in these experiments, and other experimental parameters were listed as follows: core length: 30.0 cm; section area: 4.676 cm^2^; injection rate: 0.5 mL.min^−1^, the average permeability: 1.810 um^2^. The oil displacement tests of sand-pack tube were conducted.

**Figure 3:**
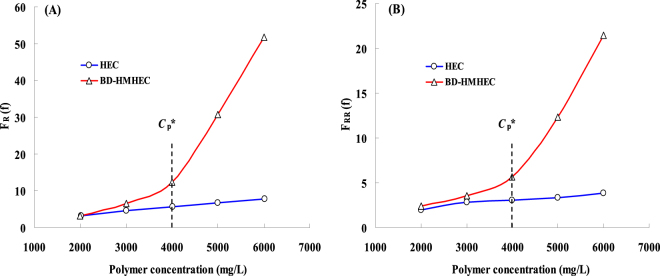
Effects of polymer concentration of BD-HMHEC and HEC on the *F*_R_ and *F*_RR_ **(A)**. The relationship between *F*_R_ and polymer concentration. (**B**) The relationship between *F*_RR_ and polymer concentration.

Figure 3 respectively shows the measured results of *F*_R_ and *F*_RR_. *C*_p_^*^ stands for the critical associated concentration. When the concentration of association polymer is blow *C*_p_^*^, infra-molecular association will occur in the polymer solution. When the concentration of association polymer exceeds *C*_p_^*^, supramolecular aggregates and hydrophobic regions are formed owing to association among the hydrophobic groups. The polymer solution has a supramolecular agglomerate structure that enlarges the hydrodynamic volume.

From Fig. 3(A,B), *F*_R_ and *F*_RR_ of BD-HMHEC and HEC both increase with the increase of polymer concentration except for some differences. When the polymer concentration is lower than the critical associated concentration (*C*_p_^*^) of 4000 mg/L, there are little differences of *F*_R_ and *F*_RR_ between BD-HMHEC and HEC solutions. But if the polymer concentration exceeds *C*_p_^*^, *F*_R_ and *F*_RR_ of BD-HMHEC begin to rise sharply, while *F*_R_ and *F*_RR_ of HEC rise slowly as before.

When the concentration of BD-HMHEC solution was below *C*_p_^*^(4000 mg/L), polymers molecules were mainly intramolecular-associated and the molecular chains tended to shrink, resulting in a smaller apparent viscosity. When the concentration reached *C*_p_^*^(4000 mg/L), the apparent viscosity of BD-HMHEC solution increased much more rapidly than that of HEC solution with increasing concentration above *C*_p_^*^. The reason is that there is a supramolecular agglomerate structure that enlarges the hydrodynamic volume at and above *C*_p_^*^ of the BD-HMHEC solution, resulting in a notable increase in the apparent viscosity value^23,34^.

#### Effect of core permeability

Core permeability is also a factor that affects *F*_R_ and *F*_RR_. In order to evaluate the effect of core permeability on the *F*_R_ and *F*_RR_, different cores with the permeability of 1.809 um^2^, 2.256 um^2^, 3.398 um^2^, 4.069 um^2^, and 5.165 um^2^ were selected in the experiments, and the other experimental parameters were listed as follows: polymer concentration: 4000 mg/L; core length: 30.0 cm; section area: 4.676 cm^2^; injection rate: 0.5 mL.min^−1^.The displacement tests were conducted.

**Figure 4:**
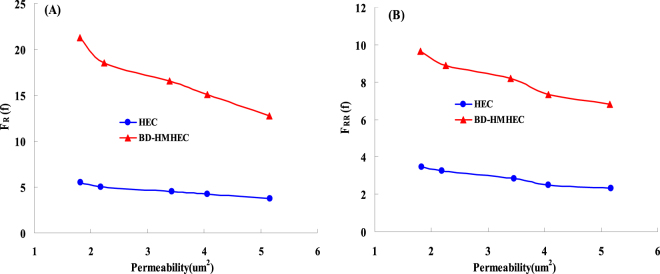
Effects of core permeability of BD-HMHEC and HEC on the *F*_R_ and *F*_RR_. (**A**) The relationship between *F*_R_ and core permeability. (**B**) The relationship between *F*_RR_ and core permeability.

Figure 4 respectively shows the measured results of *F*_R_ and *F*_RR_. From Fig. 4(A,B), *F*_R_ and *F*_RR_ both decrease linearly with the increase of core permeability. At the same core permeability, the *F*_R_ of BD-HMHEC is 12.0~21.0, which is much higher than that of HEC (6.0~10.0). Similar to *F*_R_, the *F*_RR_ of BD-HMHEC (6.0~10.0) is much higher than that of HEC (1.0~3.0).

#### Effect of injection rate

Injection rate is a chief important factor to *F*_R_ and *F*_RR_. In order to evaluate the effect of injection rate on *F*_R_ and *F*_RR_, The injection rate of BD-HMHEC and HEC solution were selected as 0.3 mL·min^−1^, 0.5 mL·min^−1^, 0.7 mL·min^−1^, 0.9 mL·min^−1^, 1.1 mL·min^−1^ respectively in these experiments, the other experimental parameters were listed as follows: polymer concentration: 4000 mg/L; Core length: 30.0 cm; Section area: 4.676 cm^2^. The displacement tests were conducted.

**Figure 5:**
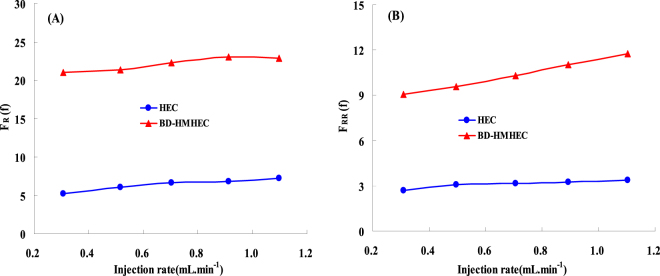
Effects of injection rate of BD-HMHEC and HEC on the *F*_R_ and *F*_RR_. (**A**) The relationship between *F*_R_ and injection rate. (**B**) The relationship between *F*_RR_ and injection rate.

Figure 5 respectively shows the measured results of *F*_R_ and *F*_RR_. From Fig. 5(A,B), *F*_R_ and *F*_RR_ both increase slightly with the increase of injection rate. Furthermore, at the same injection rate, the *F*_R_ (20.0~23.0) and *F*_RR_ (9.0~12.0) of BD-HMHEC are much higher than *F*_R_ (5.0~7.0) and *F*_RR_ (2.0~4.0) of HEC.

### Effect factors on oil displacement efficiency

In our previous study, when the temperature reached 90 °C, the nearly stable apparent viscosity value of the BD-HMHEC solution was only a small greater than that of HEC solution. However, the nearly stable value of the BD-HMHEC solution was greater than that of HEC, which illustrates that BD-HMHEC provided some improvements in the thermal-resistance performance. But, when the temperature is less than 60.0 °C, the apparent viscosity of the BD-HMHEC solution is greater than that of HEC solution. Those are attributed to the intermolecular associations due to the endothermic process of entropy increase for hydrophobic association^31^.

Block A-3 in Daqing oilfield in China was selected as an example investigate the effects on oil displacement efficiency of BD-HMHEC flooding in contrast with HEC flooding, including polymer concentration, injection slug and injection rate by a series of experiments. The actual temperature of Block A-3 in Daqing oilfield is 45.0 °C, which is less than 60.0 °C. The oil displacement efficiency of BD-HMHEC flooding would display much better than that of HEC flooding.

#### Effect of concentration

**Figure 6:**
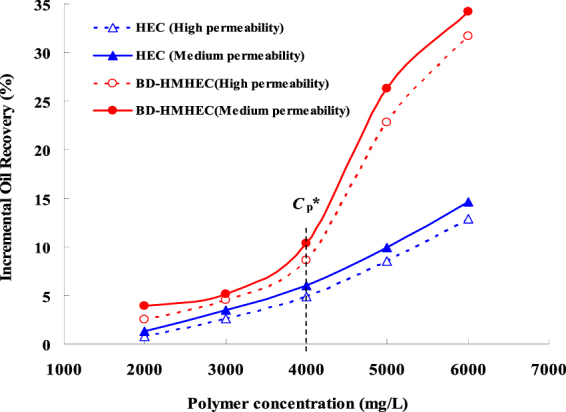
Effects on the incremental oil recovery with different polymer concentrations.

Polymer concentration affects the apparent viscosity and viscoelastic behavior which will directly affect the oil displacement efficiency. Figure 6 shows the five-couple separate experimental results of BD-HMHEC and HEC flooding varying with different concentrations (2000, 3000, 4000, 5000, 6000 mg/L) in medium (average permeability: 1.810 um^2^) and high permeability (average permeability: 5.150 um^2^) cores (core length: 30.0 cm, section area: 4.676 cm^2^; injection rate: 0.5 mL.min^−1^; injection slug: 0.5 Pore Volume (PV)).

From Fig. 6, when the polymer concentration increased, no matter whether in the medium or the high permeability cores, the incremental oil recoveries of BD-HMHEC and HEC flooding both increased monotonically and were higher in the medium permeability cores than in the high permeability cores.

When the polymer concentration was lower than *C*_p_^*^(4000 mg/L), there was little difference for the incremental oil recoveries between BD-HMHEC and HEC flooding. When the polymer concentration was above *C*_p_^*^(4000 mg/L), the incremental oil recovery of BD-HMHEC flooding increased sharply because of hydrophobic association, while for HEC flooding, it increased slightly. In a certain concentration range, the incremental oil recovery of BD-HMHEC flooding increased by 12.3~19.6% in comparison with that of HEC flooding in the medium permeability cores; while for BD-HMHEC flooding, it only increased by 9.5~18.9% in comparison with that of HEC flooding in the high permeability cores.

#### Effect of injection slug

**Figure 7:**
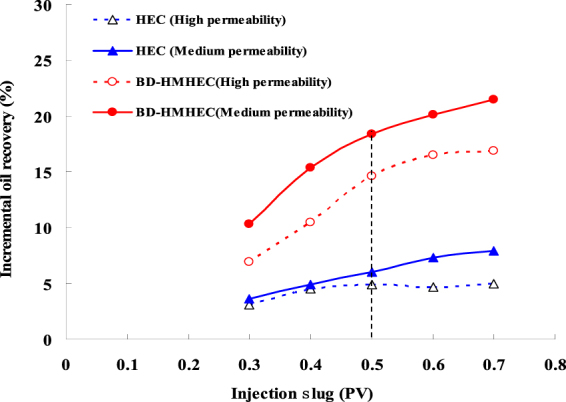
Effects on the incremental oil recovery with different injection slugs.

Injection slug directly affects the oil displacement efficiency of the polymer flooding. Figure 7 shows the five-couple separate experimental results of BD-HMHEC and HEC flooding varying with different injection slugs (0.3, 0.4, 0.5, 0.6 and 0.7 Pore Volume (PV)) in the medium (average permeability:1.810 um^2^) and high permeability (average permeability :5.150 um^2^) cores (polymer concentration: 4000 mg/L, core length: 30.0 cm, section area: 4.676 cm^2^; injection rate: 0.5 mL.min^−1^).

From Fig. 7, no matter whether in the medium or the high permeability cores, the incremental oil recoveries of BD-HMHEC and HEC flooding both increased with the increase of injection slug. For the same polymer, it was higher in the medium permeability cores than that in the high permeability cores.

When the injection slugs increased, for the medium permeability cores, the incremental oil recovery of BD-HMHEC flooding increased monotonically; while for the high permeability cores, it increased rapidly when the injection slug was lower than 0.5 PV and reached up to 14.6% at 0.5 PV, after this, the increasing rate became slower and ultimately changed to little.

As for HEC flooding, when the injection slug increased, for the medium permeability cores, the incremental oil recovery increased slightly; while for the high permeability cores, it increased at the beginning, and then gradually became stable (4.5~5.0).

#### Effect of injection rate

**Figure 8:**
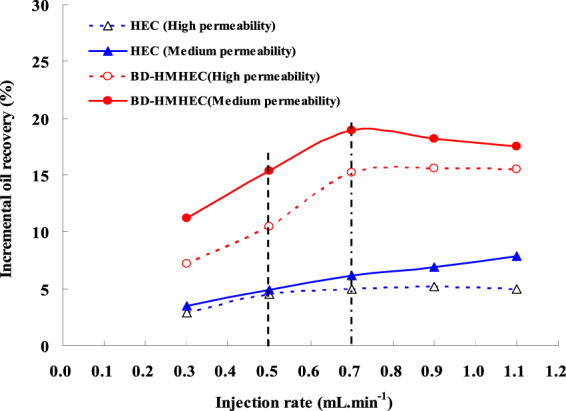
Effects on the incremental oil recovery with different injection rates.

The injection rate is an important factor that affects the oil displacement efficiency of polymer flooding. Figure 8 shows the five separate-couple separate experimental results of BD-HMHEC and HEC flooding varying with different injection rates (0.3,0.5,0.7,0.9 and 1.1 mL.min^−1^) in the medium permeability (average permeability:1.810 um^2^) and the high permeability (average permeability:5.150 um^2^) cores (polymer concentration: 4000 mg/L, core length: 30.0 cm, section area: 4.676 cm^2^; injection slug: 0.5 Pore Volume (PV)).

From Fig. 8, for the same polymer solution, the incremental oil recovery in the medium permeability core was higher than that in the high permeability core.

For the high permeability cores, when the injection rate increased, it increased rapidly up to 15.4% at 0.7 mL.min^−1^ by BD-HMHEC flooding, and then became stable; while as for HEC flooding, it changed little and when injection rate was higher than 0.5 mL.min^−1^, it became stable at around 5.0%. For the medium permeability cores, when the injection rate increased, the incremental oil recovery of BD-HMHEC flooding was similar to that in the high permeability cores. It increased rapidly at first up to 19.0% at 0.7 mL.min^−1^, and then decreased slightly, but still higher than 17.8%, while as for HEC flooding, the incremental oil recovery was different from that in the high permeability cores, it slowly increased monotonically.

### Simulation verification of oil displacement mechanism

Based on the above the studies of effect factors on oil displacement efficiency by one dimensional core displacement experiments, the simulation was conducted to verify the oil displacement mechanism. The polymer concentration (HEC and BD-HMHEC) was both selected as 4000 mg/L. The injector was injected at a constant rate (0.5 mL/min) and a constant slug (0.5PV) according to the experimental core flood tests conditions.

Other simulation parameters (such as the rock/liquid properties and producing/injecting rates, etc.) were referred to the coreflood parameters in the Block A-3 in Daqing Oilfield, China. When the flow water cut (fw) of the production liquid of the core output went into 98.0%, the simulator was terminated.

**Figure 9:**
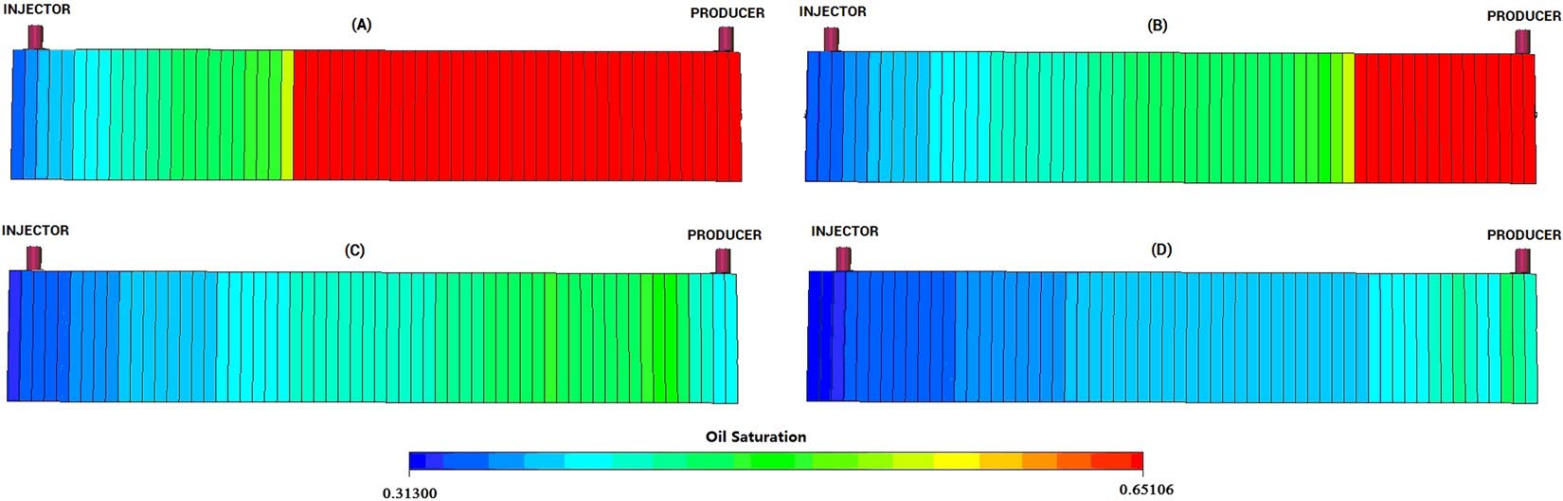
The residual oil saturation distribution of HEC and BD-HMHEC flooding (Injection slug: 0.5 PV) (**A**). HEC of the same simulated time. (**B**) BD-HMHEC of the same simulated time. (**C**) HEC with fw = 98%. (**D**) BD-HMHEC with fw = 98%.

Figure 9 shows the incremental oil recovery of BD-HMHEC flooding was improved greatly compared with that of HEC flooding. Figure 9(A,B) respectively represent the map of the residual oil saturation distribution of HEC and BD-HMHEC flooding at the same simulated time before HEC and BD-HMHEC breakthrough. Figure 9(C,D) respectively represent the map of the residual oil saturation distribution of HEC and BD-HMHEC flooding finished (when fw went into 98% after HEC and BD-HMHEC breakthrough).

**Figure 10:**
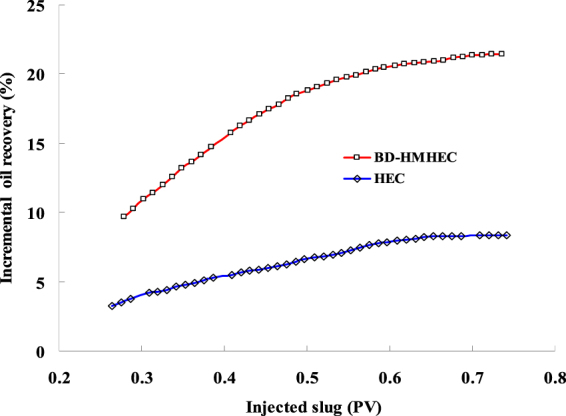
Comparison of incremental oil recovery between HEC and BD-HMHEC flooding (Concentration: 4000 mg/L).

Figure 10 represents the incremental oil recovery of simulation comparison between HEC and BD-HMHEC flooding at different injected slugs. From Fig. 10, BD-HMHEC flooding can cause better oil displacement efficiency than HEC flooding at the same condition.

### Enhanced oil recovery after polymer flooding

In order to further investigate the displacement efficiency of BD-HMHEC solution, one dimensional core displacement experiments of BD-HMHEC solution flooding following conventional polymer flooding (e.g. HPAM) and water flooding were conducted at different permeability cores.

Two small-size medium-permeability core samples (Core No. a-1#,a-2#) and two small-size high-permeability core samples (Core No.b-1#,b-2#) were selected with the section area of 4.676 cm^2^ and the length of 30.00 cm. The tests were divided into three stages: water flooding was conducted during (1) stage; HPAM flooding was conducted during (2) stage and “Switching to BD-HMHEC flooding” or “Continuing HPAM flooding” was conducted during (3) stage.

Table 1 lists the results of the continuing HPAM flooding and switching to BD-HMHEC flooding after conducting conventional HPAM/water flooding in different permeability cores.

**Table 1:** Core displacement experiments of BD-HMHEC after water flooding and HPAM flooding.

Core No.	Concentration (mg·L^−1^)		Permeability (µm^2^)	Injected slug (PV)		Incremental oil recovery (%)			
	HPAM	BD-HMHEC		HPAM	BD-HMHEC	(1) Stage	(2) Stage	(3) Stage	
						Water flooding	HPAM flooding	Switching to BD-HMHEC flooding	Continuing HPAM flooding
a-1#	1250	4000	1.915	0.5	0.5	37.9	16.9	—	2.5
a-2#	1250	4000	1.913	0.5	0.5	37.4	16.8	8.0	—
b-1#	1250	4000	5.149	0.5	0.5	54.5	12.5	—	2.3
b-2#	1250	4000	5.180	0.5	0.5	53.8	13.0	7.6	—

The results indicate that the incremental oil recovery of the HAPM was both enhanced about 12.0~17.0% after water flooding in a medium or relatively high permeability cores (after water-flooding and/or polymer flooding). But, continuing HPAM flooding improved the absolute incremental oil recovery by 2.0~2.5% after HPAM flooding; Switching to BD-HMHEC flooding can improve the incremental oil recovery by 7.0~8.0% after polymer flooding. Therefore, it is determined that the BD-HMHEC had a better oil displacement property than HPAM.

## Conclusions

The following conclusions may be drawn:

1. The effect factors on the *F*_R_ and *F*_RR_ of BD-HMHEC and HEC solution were evaluated, including polymer concentration, core permeability and injection rate. Results have shown that BD-HMHEC has higher *F*_R_ and *F*_RR_ and much better oil displacement performance than HEC.
2. The effects on oil displacement efficiency of BD-HMHEC and HEC flooding were evaluated by core tests, which show that BD-HMHEC flooding has a much better oil displacement performance than HEC flooding.
3. A numerical simulation study was performed by the CMG simulator with the different injected slugs. The results indicated that BD-HMHEC flooding can obtain better oil displacement efficiency than HEC flooding at the same condition.
4. As indicated by different core permeability tests, the incremental oil recovery of the HAPM was around 12.0~17.0% after water flooding, and the further incremental oil recovery of the BD-HMHEC was around 7.0%~8.0% after polymer flooding.
